# Apple Vision Pro Augmented Reality-Assisted Minimally Invasive Surgical Treatment of Spinal Dural Arteriovenous Fistula

**DOI:** 10.7759/cureus.63657

**Published:** 2024-07-02

**Authors:** Joshua Olexa, Kevin T Kim, Jordan R Saadon, Maureen Rakovec, Madison Evans, Jonathan Cohen, Jacob Cherian

**Affiliations:** 1 Neurosurgery, University of Maryland School of Medicine, Baltimore, USA; 2 Neurosurgery, Hoth Intelligence, Philadelphia, USA

**Keywords:** mixed reality, apple vision pro, minimally invasive neurosurgery, augmented reality (ar), spinal dural arteriovenous fistula (sdavf)

## Abstract

This report outlines the innovative use of augmented reality (AR) in the surgical planning and treatment of a spinal dural arteriovenous fistula (dAVF) via a minimally invasive technique. AR technology by way of an Apple Vision Pro headset was employed to enhance preoperative visualization and understanding of the pathology, leading to successful surgical ligation of the AVF. This case describes a 56-year-old male presenting with progressive weakness and thoracic myelopathy who showed marked improvement postoperatively, highlighting AR’s potential to improve surgical approach and outcomes.

## Introduction

Spinal dural arteriovenous fistulas (dAVFs) are the most common spinal vascular malformations, often resulting in significant neurological morbidity [1]. Surgical ligation is a definitive treatment requiring precise localization and disconnection of the fistula [2]. By superimposing virtual data onto the surgeon’s field of view, augmented reality (AR) offers an advanced tool for enhancing the precision of surgical interventions. The use of mixed reality, which includes both augmented reality and virtual reality, has grown considerably in the field of neurosurgery. Augmented reality differs from virtual reality in that augmented reality adds digital content to the user's real-world field of view, whereas virtual reality is a purely virtual experience without any visualization of the real world. New hardware and software for AR systems have enabled greater precision and potentiated new applications of this technology. More recently, the Apple Vision Pro has been released, representing a new age of mixed reality with its exceptional visual quality and powerful computational capabilities [3,4]. The Apple Vision Pro is a newly released mixed-reality headset that has already shown value in the field of neurosurgery, and it is expected that there will be growing literature describing the potential of this device to improve patient care [5]. Here, we describe the use of the Apple Vision Pro Headset for visualization and interpretation of patient-specific 3D anatomy during surgery for a spinal dural arteriovenous fistula.

## Case presentation

Case description

**Figure 1:**
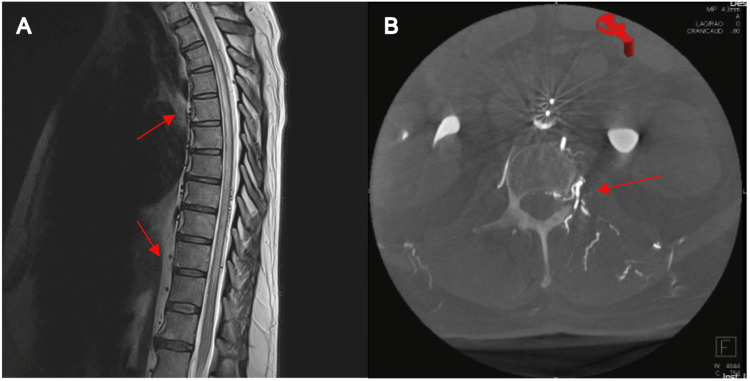
Preoperative imaging (A) T2-weighted sagittal MRI demonstrating flow voids consistent with spinal dAVF with associated T2-hyperintensity spanning central thoracic spinal cord. (B) DYNA CT completed during spinal angiogram with injection at left L2 segmental artery demonstrating type I spinal dural AVF at L2 level on the left.

A 56-year-old male presented with a six-week history of progressive lower extremity weakness, subsequent gait instability, paresthesias, and one episode of urinary incontinence. Magnetic resonance imaging (MRI) imaging of the spinal axis demonstrated prominent flow voids in the thoracolumbar spine with associated T2 hyperintense signal changes within the thoracolumbar spinal cord. Subsequent spinal digital subtraction angiography (DSA) confirmed the presence of a spinal dAVF originating from the left L2 segmental artery. Based on these MRI and DSA findings (Figure 1), the patient was diagnosed with a type I spinal dAVF at the L2 level. Given the progression and severity of his symptoms, surgical intervention was recommended.

3D model generation

A 3D model of the patient’s anatomy was generated from the DICOM imaging in 3D slicer software via a combination of manual segmentation and thresholding based on imaging intensity. In particular, the model included a 3D reconstruction of the spinal vertebrae and vasculature. The software was used to preview the model on the computer prior to uploading it to the AR headset.

Apple Vision Pro+ Software

**Figure 2:**
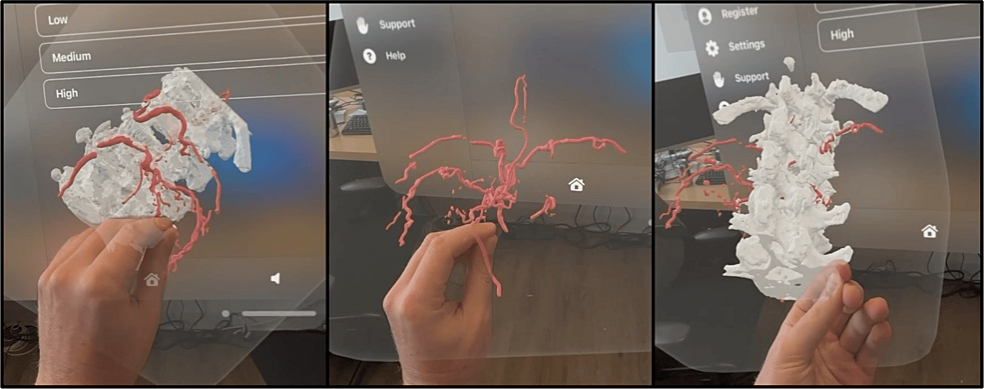
3D model viewed through the Apple Vision Pro Visualization of the patient's 3D anatomy through Apple Vision Pro. Using the software applications, users can visualize different layers to view the spinal vertebrae and vasculature either separately or together. Users can grab the 3D model with their hand to move it and enable visualization of the anatomy from different angles.

The Apple Vision Pro (Cupertino, CA) with Hoth Intelligence’s (Philadelphia, PA) software was used in anatomical visualization and surgical planning for this case. The Apple Vision Pro is a tethered video-passthrough mixed reality headset. While wearing a headset, the system displays a 3D model of the patient’s anatomy, superimposed onto the user’s real-world field of view (Figure 2).

Intervention

The patient was taken to the operating room with a surgical plan of L2 hemilaminotomy for visualization and surgical ligation of the intradural dorsal spinal AVF. Preoperative imaging data were integrated into the AR platform, providing a 3D holographic representation of the patient’s spinal anatomy and the AVF location (Video 1). The patient was intubated and placed under general anesthesia. Intraoperative neuromonitoring was used during the case, specifically somatosensory-evoked potentials, motor-evoked potentials, and lumbar-level electromyograms. The patient was then positioned prone on a Jackson table with a Wilson frame attachment in standard fashion. X-ray fluoroscopy was used to mark the location of surgery. A 5 cm incision was created, and a unilateral subperiosteal dissection was carried down to the L2 lamina under intraoperative microscope visualization. X-ray fluoroscopy confirmed the correct surgical location. Dura was exposed via an L2 hemilaminectomy and was opened to demonstrate the patient’s lumbar nerve roots as well as an obvious intradural vascular lesion of an arterialized vein. This lesion was carefully dissected and then ligated. The wound was closed in standard fashion.

**Video 1:** User experience as viewed through the Apple Vision Pro Visualization and interaction of a 3D model of spinal AVF as viewed through the mixed reality headset.

Postoperative course

**Figure 3:**
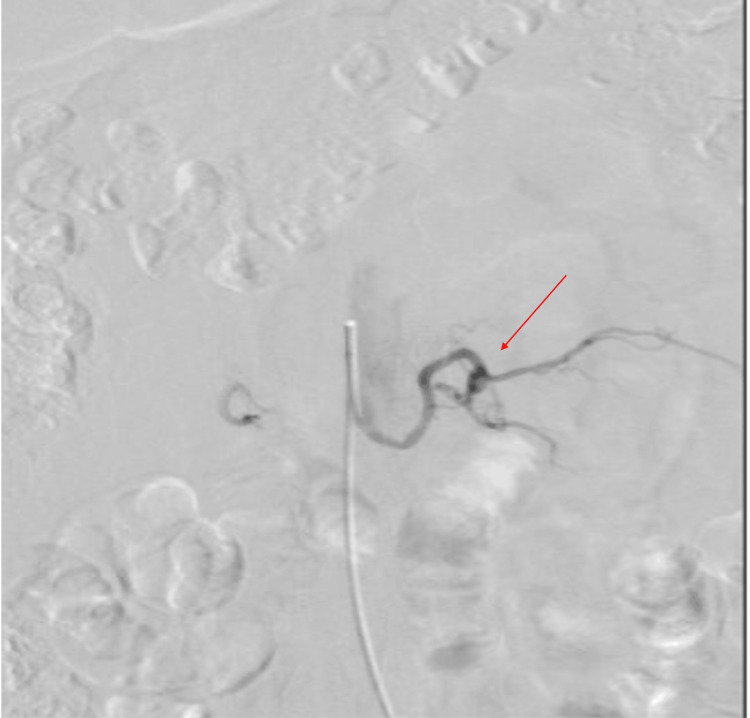
Postoperative imaging Postoperative angiogram study with injection of left L2 segmental artery demonstrating obliteration of spinal dural AVF.

The patient’s recovery was notable for a rapid and significant improvement in neurological function, with no postoperative complications. A follow-up angiogram confirmed the complete elimination of the spinal AVF (Figure 3).

## Discussion

There is a growing body of literature demonstrating the utility of augmented reality for presurgical and intraoperative planning [6-9]. An interactive and 3D understanding of patient anatomy can provide the surgical team with heightened spatial awareness of the case and offer a visual understanding that may not be appreciated with traditional 2D imaging. This case demonstrates the utility of AR technology in the surgical management of spinal dural AVFs. By providing real-time, 3D visualization of complex anatomical relationships, AR can significantly improve the surgeon’s ability to perform precise and safe dissections, allowing for minimally invasive approaches such as the case described. This report describes an early use of the latest mixed-reality headset, the Apple Vision Pro. The system utilizes advanced computation to render high-quality and seamless 3D models for the user to interact with. The computational power of the system will be critical as the field of neurosurgery demands high-detail and high-precision renderings of medical scans [3,4]. For this case, the high level of imaging detail afforded by the headset enabled the surgeon to visualize the 3D relationship between the bony vertebrae and vascular lesion.

Spinal dAVFs are a rare disease process; however, they represent the most common form of spinal AVMs [10]. Surgical management of these lesions is considered superior to endovascular treatments, with higher rates of definitive occlusion on the first treatment and lower recurrence and subsequent treatment rates [11,12]. The surgical technique for the treatment of these lesions typically consists of either a full laminectomy or a hemilaminectomy centered around the levels surrounding the nidus. Some reports describe up to three levels of laminectomy, while others describe more minimally invasive techniques. Depending on the patient’s anatomy and location of the nidus, minimally invasive approaches may be challenging for the surgeon. This case demonstrates the utility of applying AR technology to the surgical management of spinal dural AVFs. This technology was able to incorporate DYNA CT imaging from the patient’s pre-operative angiogram, providing the precise and targeted anatomic location of the nidus in relation to the patient’s bony anatomy. The technology allowed the surgeon to visualize the positioning and proximity of the dAVF to the vertebrae and thus assisted the surgical team in performing a minimally invasive approach to the lesion via a single-level hemilaminotomy window. Presurgical planning in 3D space for this case was useful for understanding these spatial relationships and demonstrated the potential application for further AR applications within microsurgical spinal techniques. Questions remain about the potential for intraoperative use of the Apple Vision Pro and augmented reality more generally. As with any new technology, further studies are warranted to assess the broader applicability of AR in spinal surgery and its long-term surgical benefits.

## Conclusions

The applications and value of augmented reality in the field of neurosurgery continue to expand. This case report demonstrates the integration of the Apple Vision Pro for presurgical planning and visualization of patient-specific anatomy in a case of spinal dural AVF. The report illustrates the ability of the Apple Vision Pro heads to provide 3D visualization of the spinal vasculature and vertebrae. By providing a more comprehensive understanding of the anatomy, AR can enhance surgical precision, reduce intraoperative risks, and improve patient outcomes, suggesting a promising future for AR in complex neurosurgical procedures. In particular, the use of the Apple Vision Pro is novel, given the recent release of this breakthrough AR device. Further studies are warranted to assess the broader applicability of AR and Vision Pro in spinal surgery and their long-term surgical benefits.
